# Draft Genome Sequence of *Klebsiella pneumoniae* Strain AS Isolated from Zhejiang Provincial Hospital of TCM, China

**DOI:** 10.1128/genomeA.00930-16

**Published:** 2016-09-22

**Authors:** Xue-Jing Yang, Sai Wang, Jun-Min Cao, Jia-Hui Hou

**Affiliations:** a Department of Clinical Laboratory, the First Affiliated Hospital of Zhejiang Chinese Medical University, Hangzhou, Zhejiang, People’s Republic of China; b Zhejiang Province Key Laboratory of Plant Secondary Metabolism and Regulation, College of Life Science, Zhejiang Sci-Tech University, Hangzhou, People’s Republic of China; c Department of Hospital Infection Control, the First Affiliated Hospital of Zhejiang Chinese Medical University, Hangzhou, Zhejiang, People’s Republic of China

## Abstract

*Klebsiella pneumoniae* is a Gram-negative, nonmotile, encapsulated, lactose-fermenting, facultative anaerobic, rod-shaped bacterium. Here we present draft genome assemblies of *Klebsiella pneumoniae* AS, which was isolated in China. The genomic information will provide a better understanding of the physiology, adaptation, and evolution of *K. pneumoniae.*

## GENOME ANNOUNCEMENT

*Klebsiella pneumoniae* is a type of Gram-negative bacterium that can cause different types of health care-associated infections, including pneumonia, bloodstream infections, wound or surgical site infections, and meningitis. *K. pneumoniae* has become an important pathogen in nosocomial infections (1). The threat of *K. pneumoniae* has increased with the emergence of strains resistant to carbapenem antibiotics (2). Here we present the draft genome sequence of one *K. pneumoniae* strain AS that was isolated from a patient in Zhejiang Provincial Hospital of TCM.

The genome of *K. pneumoniae* strain AS was sequenced using Illumina MiSeq technology at the Hangzhou Guhe Information Technology Co Ltd. (Hangzhou, China), with paired-end reads of 2 × 250 bp and an insert fragment of 450 bp. The 3,635,263 paired reads were trimmed and assembled using CLC NGS Cell v6.5. The draft genome sequence was submitted to the NCBI Prokaryotic Genome Annotation Pipeline (PGAP) for annotation (3). The annotation method was best-placed reference protein set, GeneMarkS+. Genes encoding tRNAs were determined using the tRNAscan-SE (4) and structural RNAs (5S, 16S, and 23S rRNAs) were used in a BLASTn search against the reference set. 5S hits were further refined using Cmsearch. Functional annotation was achieved using the Web CD search tool (5) and BLAST against the ARDB (Antibiotic Resistance Genes Database) (6).

The genome from *K. pneumoniae* strain AS is 5,555,747 bp long with 57.11% G+C content. After assembly, the draft genome consists of 129 contigs of >200 bp and a *N*_50_ of 95,480 bp. Overall, the AS genome has a total of 5,580 predicted features, including 5,509 coding sequences (CDS) (5,408 protein-coding genes and 101 pseudogenes), 71 RNAs (5 rRNAs, 54 tRNAs, and 12 noncoding RNAs [ncRNAs]). The top three numbers of proteins assigned to clusters of orthologous groups (COGs) are 586 (general function prediction only), 549 (amino acid transport and metabolism), 527 (carbohydrate transport and metabolism). One remarkable feature is the huge number of genes (349 genes) that are devoted to 99 kinds of antibiotic resistance types. Of these, 96 genes are annotated as MacA-MacB efflux system, which is attributed to macrolides resistance (7).

The draft genome sequence of *K. pneumoniae* strain AS determined in this study is essential for the identification of specific genetic features of this strain and for understanding the mechanisms of its antibiotic resistance activity.

### Accession number(s).

This whole-genome shotgun project has been deposited at DDBJ/ENA/GenBank under the accession no. LZCT00000000. The version described in this paper is version LZCT01000000.
